# Supplementary figures and images for: Phytosterols reverse antiretroviral-induced hearing loss, with potential implications for cochlear aging
Source: PLoS Biol. 2023 Aug 24;21(8):e3002257. doi: 10.1371/journal.pbio.3002257 (PMC10449472; doi:10.1371/journal.pbio.3002257)

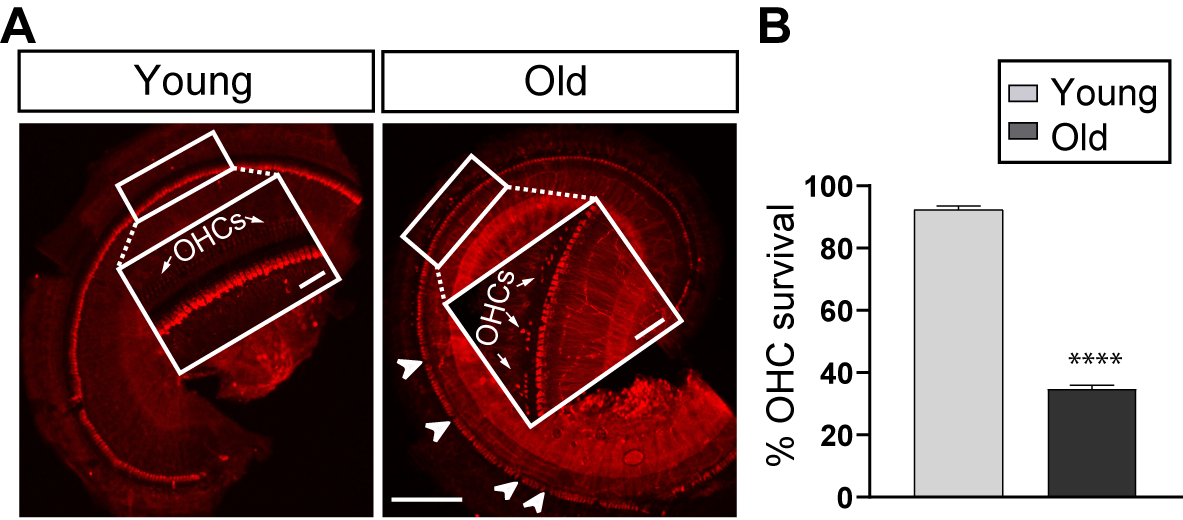

Supplement: S1 Fig — (A) Representative 10× low-magnification confocal images in the xy projection of whole mounts organ of Corti from the apical/medial region immunolabeled for calretinin from young (left; n = 5) and old (right; n = 6) C57BL/6J mice (scale bar = 200 μm). Insets: 40× magnification images of the selected area indicated by a white rectangle (scale bar = 50 μm). Arrows point to OHCs and arrowheads point to lost IHCs in aged ear tissue. (B) Bar graph showing the percentage of OHCs survival in aged mice compared to young animals. At 2 years of age, there was a 70% of OHCs death along the whole length of the cochlea. Asterisks represent the statistical significance (t test, **** = p < 0.0001). The data underlying this figure can be found at https://osf.io/xpemk/. (TIF) [file pbio.3002257.s001.tif]

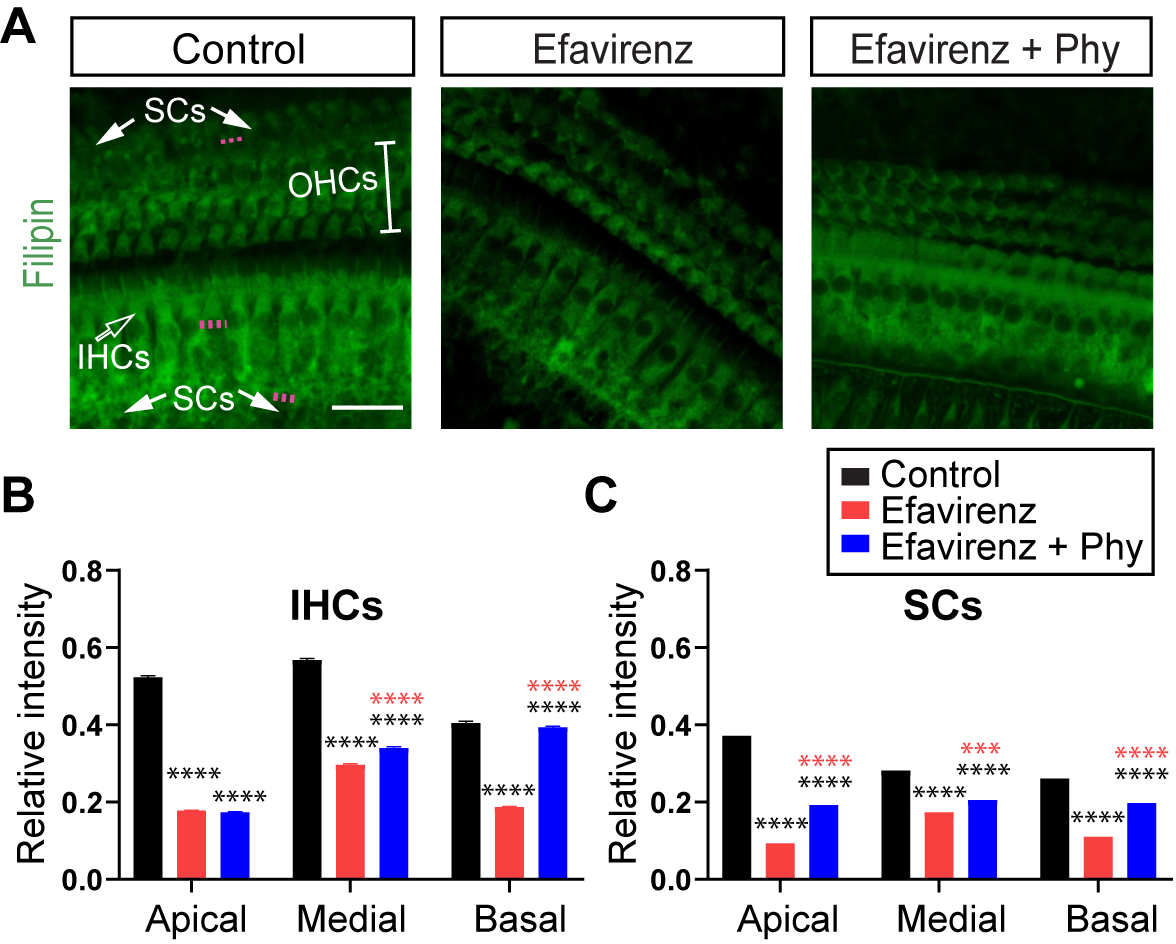

Supplement: S2 Fig — (A) Representative confocal images of whole mounts organ of Corti from the medial region stained with filipin in control, treated with efavirenz, and treated with efavirenz plus Phy mice. (Scale bar = 30 μm). Pink lines indicate the diameter of IHCs and SCs as an example on how we select cells to measure the pixel intensity. (B) Relative intensity bar graph showing the changes in filipin staining in IHCs in the 3 groups of mice at the 3 regions of the cochlea (apical, medial, and basal) (Control: n = 48 IHCs at the apical, 128 IHCs at the medial, and 66 IHCs at the basal region from 6 mice; efavirenz: n = 81 IHCs at the apical, 75 IHCs at the medial, and 35 IHCs at the basal region from 6 mice and efavirenz together with phytosterols: n = 93 IHCs at the apical, 114 IHCs at the medial, and 60 IHCs at the basal region from 6 mice). (C) Relative intensity bar graph showing the changes in filipin staining in SCs in the 3 groups of mice at the 3 regions of the cochlea (Control: n = 10 SCs at the apical, 21 SCs at the medial, and 10 SCs at the basal region; efavirenz: n = 22 SCs at the apical, 12 SCs at the medial, and 11 SCs at the basal region and efavirenz together with phytosterols: n = 14 SCs at the apical, 11 SCs at the medial, and 10 SCs at the basal region). There was a significant reduction in cholesterol levels after efavirenz treatment in both IHCs and SCs. Treatment with efavirenz together with Phy showed an increase in filipin staining at the 3 regions compared with mice treated with efavirenz alone, except in IHCs at the apical region. However, when compared to controls, there was a significant reduction in filipin staining at the 3 regions of the cochlea in both IHCs and SCs. Group means ± SEM are shown. Asterisks represent the statistical significance (one-way ANOVA, followed by Tukey’s test, = *** p < 0.001, **** = p < 0.0001). The data underlying this figure can be found at https://osf.io/xpemk/. (TIF) [file pbio.3002257.s002.tif]
