# Supplementary material for: Phytosterols reverse antiretroviral-induced hearing loss, with potential implications for cochlear aging
Source: PLoS Biol. 2023 Aug 24;21(8):e3002257. doi: 10.1371/journal.pbio.3002257 (PMC10449472; doi:10.1371/journal.pbio.3002257)
Supplement: Attachment — Submitted filename: Response to Reviewers.docx [file pbio.3002257.s003.docx]

**Revision**

**Title: "Phytosterols reverse hearing impairments caused by antiretroviral treatment. Implications in presbycusis"**

**PBIOLOGY-D-22-01971R1**

**Authors: Alejandro O. Sodero, Valeria C. Castagna, Setiembre D. Elorza, Sara M. Gonzalez-Rodulfo, María Alejandra Paulazo, Jimena A. Ballestero, Mauricio G. Martin and María Eugenia Gomez-Casati**

**Editor's Remarks to Author:**

***As you will see in the reviewer reports, which can be found at the end of this email, although the reviewers find the work potentially interesting, they have also raised a number of important concerns, which would need to be thoroughly addressed before we can consider your manuscript further for publication. Having discussed the reviews with the Academic Editor, it is clear that a substantial amount of work would be required to expand and strengthen the mechanism provided here, which we think would be essential in order for this study to meet the criteria for publication in PLOS Biology.***

***However, given our and the reviewer's interest in your study, we would be open to inviting a comprehensive revision of the study that thoroughly addresses all the reviewers' comments, with new analyses and data. To this end, we are willing to relax our standard revision time to allow you 6 months to revise your study.***

We would like to thank the PLOS Biology Editors and the Academic Editor for giving us the opportunity of revising our work and the reviewers for their helpful comments.

We have exhaustively reworked the manuscript to fully address the reviewers’ suggestions, in order to expand and strengthen the conclusions provided in the present work. The manuscript has been changed accordingly and a point-by-point response to major and minor concerns raised by the three reviewers is included. We have now introduced a detailed description regarding the significant conceptual advance that our work provides in the field of hearing research.

In the present work we show that: 1) aging triggers cholesterol loss from sensory cells of the inner ear, 2) a retroviral treatment widely employed for HIV/AIDS patients, reproduces the cholesterol loss observed in aged individuals and leads to impaired outer hair cells’ function and 3) we found that these defects can be partly reversed by phytosterols supplementation. Our findings are very promising because they provide the first proof-of-principle supporting phytosterols supplementation as a possible approach for prevention or treatment of hearing loss.

Thank you very much for your consideration.

Sincerely,

María Eugenia Gómez-Casati and Mauricio G. Martin

**Reviewer Comments:**

**Reviewer #1:**

***In the manuscript 'Phytosterols reverse hearing impairments caused by antiretroviral treatment. Implications in presbycusis.' Sodero et al., describe an increase in CYP46A1 leads to a decrease of cholesterol in the outer hair cells of aged mice and mice treated with an antiretroviral drug used in HIV/AIDS treatment. They are able to show that administering this drug in young mice affects outer hair cell function as measured via DPOAEs and can be partially rescued by feeding phytosterols to the same animals. In general, I think this is a very interesting study that is of interest for hearing research. However, I do have some concerns with this study.
1)      Fig1A: The calretinin stain is very hard to see in the OHC in the young group, but very clear in the aged group. Since it is reported (e.g. Dechesne CJ, Rabejac D, Desmadryl G. Development of calretinin immunoreactivity in the mouse inner ear. J Comp Neurol. 1994 Aug 22;346(4):517-29. doi: 10.1002/cne.903460405. PMID: 7983242.) that calretinin is not expressed in the outer hair cells in the adult (>P22) that is very surprising to me. Are you sure those are OHCs?***

We thank the reviewer for acknowledging the fact that our work is an interesting study for the hearing research community.

The reviewer is right that calretinin stain is very hard to see in the OHCs in the young group, but very clear in the aged group. We performed a search in the literature and in the Gene Expression Analysis Resource database (gEAR portal) and found that calretinin is indeed expressed, although very weakly, in young adult OHCs. Using RNA-seq from young adult CBA/J mouse cochleae, the lab of He found a weak expression in OHCs compared to IHCs (Liu *et al.,* 2018; PMID: 30327589). Immunohistochemical studies in mice at postnatal day 20 (P20) showed also a weak expression of calretinin in OHCs using the same anti-calretinin antibody as in present study (Savoy-Burke *et al*., 2014; PMID: 25264928; Pyott *et al*., 2004; PMID: 15509733).

It is important to note that we used a different anti-calretinin antibody as in the paper mentioned by the reviewer (Dechesne *et al*., 1994; PMID: 7983242.). Thus, the low level of expression of calretinin together with the use of different antibodies could be the explanation of the discrepancy seen in the labeling in our study in 2-month-old animals compared to Dechesne *et al*. 1994 (i.e. a very week labeling versus no labeling in young adults, respectively).

In our whole mounts immunostaining using anti-calretinin antibody we observed an increase in the labeling in those surviving OHCs in mice at two years of age. Similarly, recent studies on cell type-specific transcriptomic analysis on aged mouse cochlear inner and outer hair cells, showed an increase in the expression of calretinin (*Calb2* gene) specifically in OHCs at 26 months of age (Liu et al., 2022; PMID: 35417713). We have now included a Supplementary Figure 1 showing low magnification images of calretinin labeling in the apical/medial region of the cochlea in young and aged mice. In aged animals, it is clear the positive labeling in the OHCs’ region. It has been shown that the calcium-binding protein calretinin plays a critical role in buffering intracellular calcium during cellular stress in all cochlear nuclei (Lohmann and Friauf, 1996; PMID: 8867285) and in brain aging (Vishnyakova *et al*., 2021; PMID: 33040447; Ahn *et al*., 2017; PMID: 28944879). In several brain regions like the posteroventral cochlear nucleus (PVCN), and dorsal cochlear nucleus (DCN) there was an increase of calretinin expression in aged mice compared to young animals (Elliott at al., 2022; PMID: 35250542) and this increase in the calcium binding protein was shown to be correlated with hair cell loss (Idrizbegovic et al., 2003; PMID: 12742236; Caspary and Llano, 2018). We have now described these observations in the Results and Discussion sections of the modified version of the manuscript.

In the new Supplementary Figure 1, we also included a bar graph showing the percentage of OHCs survival in aged mice compared to young animals. At two years of age, there was a 70% of OHCs death along the whole length of the cochlea. We counted the OHCs along the whole cochlear sections combining the calretinin positive signal together with light microscopy using Nomarski optics, based on the assessment of all present and absent hair cells in all sections. Representative pictures also showed that at two years of age there were changes in IHCs morphology together with some IHCs loss (arrowheads in Fig S1. point to lost IHCs in aged ear tissue).

***2)      The animal numbers are not completely clear to me. Did you use the same animals for the different immunostainings? Why were there only 3 animals for the filipin staining in the young group but 5 for the CYP46A1 staining in the same age group?***

We used different animals for the different immunostainings in the young group. For the CYP46A1/calretinin whole mount immunostaining we used 5 animals (10 cochleas) and for the filipin/phalloidin labeling we used 3 animals (6 cochleas).

***3)      While the authors do mention that C57BL/6 mice are known to lose their hearing early it would be beneficial to discuss why they chose this mouse strain anyways vs a mouse strain like CBA/CaJ which are known to retain their hearing up to an old age. In that case the conclusion of age being the driving factor of the reduction and redistribution of cholesterol in the OHCs would have had much more merit. In the present study it is hard to prove that this effect is due to age and not also influenced by the overall damage to the OHCs. This should be discussed.***

The reviewer is right that performing the experiments in another mouse strain, like CBA/CaJ or BALB/cJ, would have been much better because of the already well described early onset of age-related hearing loss in the C57BL/6J strain. When we planned to start this project in the cochlea, we already had aged C57BL/6J animals reserved for experiments in the central nervous system (CNS). Among the strains, the C57BL/6J is the most widely used for aging studies since these mice are able to survive longer (with the maximum being 27 – 29 months). Indeed, our previous studies about alterations of cholesterol homeostasis in aged mice were performed in C57BL/6J mice. Using this strain, we have shown that aging increases CYP46A1 levels in the hippocampus of 24 month-old mice (Martin et al., 2014; PMID: 24878762) leading to cholesterol loss and cognitive decline. These deficits, at least in the hippocampus, are not observed before 19 months of age. That was the reason for exploring the CYP46A1 levels at an advanced age in C57BL/6J mice. However, the results previously obtained in the hippocampus do not necessarily imply that the same defects would occur earlier in hair cells of the CBA/CaJ or BALB/cJ strains. We need to analyze CYP46A1 levels in hair cells at earlier times (i.e. 12 months) and for that it would be possible to use CBA/CaJ or BALB/cJ mice. In any case, to avoid that hearing defects developed by C57BL/6J mice starting at 8-10 months of age would influence our conclusions, we performed the efavirenz and efavirenz plus phytosterols studies starting the treatment at 2 months of age to avoid the auditory threshold elevations with age described for this strain (Someya S. *et al.*, 2009; PMID: 19901338).

As the reviewer mentioned, in fact, we already started a project on aging BALB/cJ mice (until 1 to 2 years) to analyze if phytosterols supplementation can delay the onset of presbycusis. Nevertheless, we consider that our present work is very important because it is the first proof-of-principle to show that CYP46A1 activation can lead to hearing deficits because of its action in cholesterol removal from OHCs, leading to alterations in the distribution of the motor protein prestin with functional consequences. Moreover, we show that the effect of cholesterol loss on this protein localization on OHCs can be rescued with phytosterols supplementation in the diet. As the reviewer suggested, we have now added this issue in the Discussion section in the new version of the manuscript.

***4)      In the aging study it is mentioned that an increase in CYP46A1 and decrease in filipin staining can be observed not only in OHCs but also IHCs and SCs. Was that also the case with the drug treatment?***

As the reviewer suggested, we analyzed the filipin staining in IHCs and SCs in controls, efavirenz-treated and supplemented with phytosterols groups and now show the results in the new Supplementary Figure 2.

***5)      Fig 1A: the merged of the aged animals does not line up with the Calretinin only stain.***

The merged figure now is lined up with the calretinin only stain. This has been corrected in the revised version of the manuscript.

***6)      Fig 4A: in the Efavirenz+Phyto group it seems as if Filipin is located to the cell membrane vs in the other groups where it is more central. Also, the Phalloidin intensity is reduced in this group compared to the others. And in the Efavirez group it seems to be more diffusely distributed. Please explain this.***

Filipin detects cholesterol and phytosterols which are mainly enriched in detergent resistant domains of cell membranes, including intracellular membranes (Vanmierlo et al., 2012; PMID: 22279184). Thus, it is expected that filipin stains the whole cell. Although the filipin distribution seems to be different in some cells, the quantification of the filipin intensity along a high number of cells in the different groups shows a similar distribution. This can be appreciated in Figure 4B.

Phalloidin is a highly selective bicyclic peptide used for visualizing actin in stereocilia of cochlear hair cells. The reviewer is right that in the representative pictures in Figure 4A it seems that phalloidin intensity is reduced in the Efavirenz + Phyto group compared to the others. To analyze the phalloidin staining in all the groups, we performed a quantification analysis of fluorescence intensity in OHCs. We found no difference in the phalloidin intensity in the different sections of the three groups (*p* > 0.05 ANOVA).

The phalloidin pixel intensity (arbitrary units) in the OHCs of the different groups was the following:

**Control** (mean ± SEM): Apical 1023 ± 13.13; Medial 1364 ± 8.98 and Basal 1289 ± 11.79. **Efavirenz** (mean ± SEM): Apical 1027 ± 7.21; Medial 1355 ± 8.70 and Basal 1297 ± 11.83. **Efavirenz + Phy** (mean ± SEM): Apical 1020 ± 6.04; Medial 1369 ± 17.77 and Basal 1265 ± 8.43.

We have now included this information in the results section and we have also changed the picture for more representative ones.

***7)      I am not sure about the reasoning of using the average of either two or three OHCs for the analysis in Figures 4 & 5.***

To visualize cholesterol in the OHC lateral wall, we used filipin labeling of mouse OHCs. In Figure 4, we analyze filipin pixel intensities by drawing a line through the middle of two consecutive OHCs, and then plotting them as both pixel intensities versus distance and relative intensity bar graphs at the different cochlear regions. It is important to clarify that although we draw the line for the pixel intensity analysis in groups of two consecutive OHCs, we analyzed all the OHCs we imaged from each confocal z-stack. The pink bar in the Figure 4 represents 16 μm, being 8 μm the average diameter of a single OHC. We choose to select two consecutive OHCs in this analysis because we wanted to be sure that the line is right in the middle of the cells, so we can analyze the same region of the cells. For this reason we could not select more than two OHCs. We performed this analysis according to what was previously published by (Rajagopalan et al., 2007; PIMD: 17933870) except that in their images they analyzed them by selecting individual OHCs. For comparison, we performed the same analysis by selecting individual OHCs and we obtained the same results as selecting them in groups of two consecutive cells.

In the case of Figure 5, the whole mounts organ of Corti immunolabeled for prestin, we chose to draw a line through the middle of one (Figure 5B and C) or three (Figure 5D) consecutive OHCs. Due to the fact that prestin labeling is more robust than filipin and we could visualize the whole length of the OHC, in this case it was easier to pick three consecutive cells that are in the same plane. In the new version of the manuscript, we have now changed the prestin intensity plot versus the distance from three OHCs to one individual OHC (new Figure 5B) to better visualize the changes in the lateral wall membrane of the cell in the three groups of mice. In conclusion, we analyze all the OHCs we imaged from each confocal z-stack by drawing the line in groups of one, two or three consecutives OHCs.

We clarified this issue in the Results and Materials and Methods’ section in the revised version of the manuscript.

***8)      Is there a reason why you represent different areas in the cochlea for your filipin vs prestin stain intensity?***

We dissected the whole cochlear spiral epithelia and performed the analysis at three different locations: apical (encoding low-frequency sounds), medial, and basal (encoding high-frequency sounds). The reason we did that is because we wanted to discern if there is a gradient of filipin and/or prestin labeling along the different frequencies of the cochlea. The spatial separation of sounds based on frequency (tonotopy) is one of the most fundamental principles of auditory function. It has been shown that tonotopic expression gradients exist for several proteins, growth factors and molecules along the apical/basal axis of the mammalian cochlea. Thus, it is very important to analyze the different regions of the cochlea for identification of the molecular cues controlling the tonotopic processes in the sensory epithelium.

Here, we found that at all the regions of the cochlea there was a difference in both filipin and prestin labeling. In Figures 4A-B and 5A-C, we selected the best representative maximum projection z-stacks pictures with their corresponding pixel intensity graph, which plots pixel intensities from all the ear sections along the length of two or one OHC at the same region, respectively (i.e. Basal region in Figures 4A-B and Medial region in Figures 5A-C). Nevertheless, in Figures 4C and 5D, we show the pixel intensity bar graph showing the changes in filipin and prestin staining, respectively, in the three groups of mice at all the regions of the cochlea (apical, medial and basal).

***9)      How did you select the two/three OHCs to measure across?***

For the quantification of OHCs’ cholesterol concentration by filipin staining and for the quantification of prestin fluorescence intensity, we draw a line through the middle of two and one/three OHCs, respectively. The pink/pink-orange bars in both Figures 4 and 5 represent an example of how we select the groups of cells. It is important to make clear that we quantify all the OHCs in each confocal z-stack from each section in groups of two (filipin staining) or one/three (prestin immunostaining) in each case.

We have now clarified this in the new version of the manuscript.

**Minor Comments:**

***1) Please use the same citation format throughout the manuscripts.***

We have now unified the citation format throughout the whole manuscript.

***2) In the table with the questions from the journal you state that an ethics approval was not necessary for this study despite conducting an animal study (which was listed as one of the reasons to include an ethics statement).***

We have now changed that and include the ethics committee’s approval letter.

***3) It is not always clear if the pink arrow heads are considered part of the line or if it is only the distance between the two arrow heads that is marked.***

The pink arrow heads are indeed part of the line. But, to avoid confusion we have now changed it for a line only without arrow heads.

***4) In the results section you write that you treat for 3 weeks with the phytosterols. In the methods section it is stated that the treatment was 4 weeks. Please correct where necessary.***

Thanks for noticing that. We have now corrected that the treatment with the phytosterols was for 3 weeks in the new version of the manuscript.


**Reviewer #2:**

***Sodero et al. studied the role of cholesterol in hearing loss by analyzing the relationship between the decrease in cholesterol levels and outer hair cell degeneration. They detected elevation of DPOAE thresholds after administration of the mice with efavirenz, an antiretroviral which pharmacologically activates an enzyme responsible for cholesterol turnover (CYP46A1). Interestingly, the elevation in DPOAE thresholds due to efavirenz administration was prevented by dietary complementation with phytosterols. If this observation holds true, it would be of high interest as a strategy for preventing hearing loss due to age-related issues and ototoxic drugs. However, there are some important concerns about these claims since the experiments do not provide a clear mechanism of action. Also, there is no elevation of auditory thresholds (ABRs) caused by the drug administration, which dampens enthusiasm for work.   
Some important points to consider:
-       Can the authors use qRT-PCR for quantifying CYP46A1 levels? Immunostaining can be highly variable across experiments. Is the difference in CYP46A1 quantity between young and old mice stronger in the base or the apex? Only mid sections were used in Fig 1.***

We thank the reviewer for acknowledging the fact that our work is of high interest as a strategy for preventing hearing loss due to age-related issues and/or ototoxic drugs.

We recognize that the immunostainings can show variations across experiments, but it is the best technical approach to analyze the regional expression of CYP46A1 in the cochlea instead of using quantitative PCR in whole mount tissue that does not allow us to study the specific cellular expression of this enzyme.

As the reviewer suggested, we performed a new quantification analysis of CYP46A1 immunofluorescence intensity levels at three regions of the cochlea: apical, medial and basal. We observed that the difference in CYP46A1 levels between young and old mice is stronger in the mid-base region of the cochlea compared to the apex. We have now included the CYP46A1 fluorescence intensity plots of young and old animals at the three cochlear regions in the new Figure 1B.

Moved by the reviewer's suggestion, we also measured the CYP46A1 mRNA levels using a pool of young (2 months, n = 8) and middle-aged (12 months, n = 11) cochleas. Due to the small tissue size it was not possible to work with mice individually, so we needed to pool several cochleas to generate each time point. Unfortunately, in the 6-months period of this revision, we did not have available mice of 24 months of age in our animal facility to generate an “old” time point. We were able to purify total RNA using retention columns, to retro-transcribe 450 ng of RNA, and perform a SYBR Green-based quantitative PCR analysis in triplicates. We used GAPDH as a housekeeping gene. We found that the relative expression levels of CYP46A1 in the inner ear were similar in young and middle-aged mice. This result was similar to that previously reported in the aging hippocampus (Martin et al., 2008; PMID: 18287532). It is worth clarifying that a large number of CNS changes induced by aging in C57BL/6 mice start to be evident once the animals reach the 22 months of age (as example see Lemaitre et al., 2023; PMID: 36975362). This is the reason why the usual experimental time point for aged C57BL/6 mice is 22-24 months.

***-       Could you explain how were the ROIs defined for the quantification of CYP46A1 in the supporting cells?***

For the quantification of CYP46A1 fluorescence intensity in the supporting cells (as well as sensory cells), the region of interest (ROI) was settled by the cell boundaries of those fluorescently-labeled cells. This was done by selecting the boundary of each cell and adding each one to the ROI Manager: Analyze / Tools / ROI Manager on the Fiji software. It is important to note that we did not discriminate between the different types of supporting cells. We have now added this information in the Materials and Methods’ section and labeled some cells, as an example, in the new Figure 1A of the revised version of the manuscript.

***-       In Fig 2, since there is substantial hair cell loss in aged mice, the orientation of the cells is less consistent which makes it difficult to compare with younger mice that have a better-preserved Organ of Corti. How was the IHC cholesterol concentration quantification performed? In which part of the cell body was the analysis done?***

The reviewer is right that because of the substantial OHCs’ loss in aged mice, it is difficult to see the tissue orientation. We have now clearly labeled the different cell types in the new Figure 2.

For the quantification of IHCs’ cholesterol levels, as we did for OHCs, we draw a line through the middle of every individual IHC in the image and fluorescence intensity was quantified along this line. This is shown as a pink dotted line in the new Figure 2. We have now clarified this in the Materials and Methods’ section in the new version of the manuscript.

***-       Is the effect of efavirenz (and phytosterols) dose dependent? Could higher doses of the drug cause ABRs elevations? The fact that the DPOEAs thresholds are elevated with no effect on ABRs is surprising because OHC function is necessary to maintain normal ABR levels, and also this indicates that there is no evident hearing loss associated with efavirenz administration.***

We do not know if the effect of efavirenz (or phytosterols) in the inner ear is dose dependent in our *in vivo* system.

A previous work has found both in isolated brain microsomes and *in vivo*, that the efavirenz effect on CYP46A1 was dose- and time-dependent. The dependence of CYP46A1 activity on efavirenz concentration represented a bell-shaped curve with CYP46A1 activation at low efavirenz concentrations and inhibition at higher drug concentrations. Low doses of efavirenz stimulated cerebral cholesterol turnover and this mechanism is probably allosteric with efavirenz binding outside the CYP46A1 active site. At higher doses, efavirenz inhibited the CYP46A1 enzyme and its mechanism is probably based on a competition between the substrate (cholesterol) and efavirenz for the P450 active site (Mast N. *et al*., 2014; PMID: 24352658).

Here, we observed that ABR thresholds after efavirenz treatment in C57BL/6J mice were slightly elevated (see representative ABR responses in the new Figure 3A), although it was not statistically significant using Kruskal–Wallis nonparametric ANOVA followed by Dunn’s post-test that analyzes the three experimental groups: Control, Efavirenz and Efavirenz + Phytosterols. We think that this mild, but not statistically significant, ABR threshold elevation after efavirenz treatment is due to the OHCs dysfunction. It is important to note that DPOAEs threshold elevation is not very large, approximately 20-30 dB, and it is only statistically significant at three mid frequencies. Thus, if DPOAEs threshold elevation is not very large it is expected that ABR elevations are not very large as well. We have now included in the new version of the manuscript ABR representative traces in the three conditions in the new Figure 3 for better appreciation of the mild ABR threshold elevation in mice treated with efavirenz.

It is important to remark that Kruskal–Wallis nonparametric followed by Dunn’s post-tests is the most widely used test to determine statistical significance in this case where data are non-normally distributed and we are comparing three different groups: Control, Efavirenz, Efavirenz + Phytosterols. However, if we analyze controls and efavirenz-treated groups by using Mann Whitney u-test the ABR thresholds elevation are significant at 16 and 32 kHz (p *<0.05).

Considering the already described dose-dependency of efavirenz, potentiation at low doses and inhibition at higher concentrations, we agree with the reviewer that it is important to test if higher doses of the drug could produce higher auditory threshold elevations.

Due to the fact that we did not have enough C57BL/6J mice at our animal facility, we performed the experiments in the BALB/c strain. Thus, we treated 2-month old BALB/c mice with efavirenz in the drinking water at two different concentrations for 4 weeks:

1) **Efavirenz Low** = 0.09 mg/kg/day (the same concentration used in the experiments with C57BL/6J mice) and
2) **Efavirenz High** = 0.18 mg/kg/day.

After 4 weeks of treatment, we measured the auditory function by ABRs and DPOAEs. As shown in the Figure 1 below, we found no significant changes in both ABRs thresholds and P1 amplitude at low and high efavirenz dose. However, when we recorded DPOAEs we observed a slight threshold elevation at low efavirenz concentration that was significant only at 11.33 kHz using Mann Whitney u-test (p *<0.05). At high efavirenz dose there was no difference in DPOAEs thresholds compared to controls.

**Figure 1**: Auditory function in control (black; n = 8), efavirenz low (0.09 mg/kg/day; red; n = 8) and efavirenz high (0.18 mg/kg/day; green; n = 9) BALB/c treated mice. Mean ABR thresholds (**A**), ABR peak 1 amplitudes at 80 dB SPL (**B**) and DPOAEs thresholds (**C**) at different test frequencies. At the moment of the auditory recordings mice were 12 weeks old. DPOAEs thresholds showed a significant increase only at 11.33 kHz in those mice treated with efavirenz at the lowest concentration. Group means ± SEM are shown. Asterisk represent the statistical significance (Kruskal–Wallis test, * = p<0.05).

Thus, efavirenz at a low dose (0.09 mg/kg/day) in BALB/c mice had a smaller effect compared to the same concentration in C57BL/6J mice. It could be possible that 0.09 mg/kg/day (Efavirenz low) is not the proper concentration to potentiate the CYP46A1 enzyme, and a lower dose is required in BALB/c mice. *In vitro* studies on the mechanism of CYP46A1 activation showed that 20 μM efavirenz was the maximal concentration for CYP46A1 activation, with a subsequent decrease in the CYP46A1 activation and ultimately enzyme inhibition as the efavirenz concentrations were increased (Mast N. et al., 2014; PMID: 24352658). Moreover, it has been observed that enzyme activation depends on the ratio between the CYP46A1 protein, the substrate (cholesterol), and the activating drug (efavirenz) (Mast N. et al., 2014; PMID: 24352658). Thus, the weaker CYP46A1 activation in BALB/c mice compared to C57BL/6J mice could be probably due to a different ratio between CYP46A1, cholesterol and efavirenz in the different tested mouse strains.

In this new set of experiments in BALB/c mice we also observed that if we increased the dose of efavirenz to the double (Efavirenz High = 0.18 mg/kg/day) both ABRs and DPOAEs are indistinguible from controls and this could indicate that instead of potentiating we are blocking CYP46A1 activity compared to the Efavirenz Low group.

Another possibility is that we may need to increase the treatment time in the BALB/c strain to obtain a higher CYP46A1 activation.

In conclusion, in order to analyze the dose dependency of efavirenz we will need to test first different concentrations of efavirenz in C57BL/6J mice and then test different protocols of efavirenz treatment like for example: testing longer times, different concentrations or even a different delivery method (like gavage). We are willing to analyze more deeply the dose dependency in different mouse strains as part of coming projects.

-       ***Could the authors perform ABR recordings of the treated mice at 2 months of age prior to the drug treatments? Then, comparisons can be made within each animal.***

The C57BL/6J mouse strain, which is widely used for aging research, displays the classic pattern of age-related hearing loss starting at 8-10 months of age (Someya S. *et al.*, 2009; PMID: 19901338). The onset of age-related hearing loss in this strain of mice begins in the high-frequency basal region of the cochlea and spreads toward the low frequencies with age. These functional deficits are accompanied by the loss of hair cells and neurons.

We could not perform ABR recordings of the treated mice at 2 months of age prior to the drug treatments. We had two reasons for not doing that: 1) the ABR/DPOAE acoustic chamber is in our laboratory, and we are not allowed to take the animals out of the animal facility for experiments and then back to the animal room for drug treatment and 2) for ABR/DPOAE recordings we need to anesthetize the mice with an intraperitoneal injection of ketamine/xylazine and this might have an impact on water and food intake, that could affect the efavirenz and efavirenz + phytosterols treatments.

In any case, it is well established that C57BL/6J mice at 2 months of age have a good hearing sensitivity by ABR and DPOAEs recordings (Kikkawa Y. et al., 2012; PMID: 22531723; Parham K., 1997; PMID: 9367243). Nevertheless, we have now included ABR recordings in C57BL/6J mice at 2 months (8 weeks) as control. As it is now shown in the new Figure 3A, at 8 weeks of age, ABR thresholds were the same as the control group (i.e. at 12 weeks of age (8 weeks + 4 weeks of treatment).

***-       Line 407-409: "Studies in mice suggested a synergistic relationship between anti-HIV drugs and noise, with an increase in DPOAE thresholds consistent with drug-induced damage to OHCs" Would the efavirenz-treated animals be more sensitive to noise-exposure?***

In the study by Bektas, D. *et al* 2009; PMID: 18384985, the authors examined the effects of anti-HIV drugs combined with intense sound exposures on auditory function in mice. They perform both ABR and DPOAEs measurements in mice treated with high-dose combinations of anti-HIV drugs. Although the anti-HIV drug combination that they used did not include efavirenz, their results showed no differences in either DPOAEs or ABRs between the drug-treated experimental and control groups. However, they observed that after exposure to high intensity noise DPOAEs thresholds did not recover in the drug-treated mice.

We did not test if efavirenz-treated animals are more sensitive to noise-exposure, and it will be an excellent idea to test in our new set of experiments in BALB/cJ mice designed to study the dose and/or time dependence of efavirenz treatment.

***-       Can you show the filipin staining of IHC after efavirenz (and Phy) treatment (Fig 4)?***

As the reviewer suggested, we have now added a new Supplementary Figure 2 which includes the filipin staining in both IHCs and SCs in the three groups conditions together with their quantification analysis.

-       ***Are the newly delivered phytosterols being incorporated into the membrane? Can you radiolabel the molecules and do a quantitative analysis of these lipids incorporation?***

We agree with the reviewer on the proposed experiment using radiolabeled phytosterols to unequivocally confirm the incorporation of these molecules to the plasma membrane but unfortunately none of the participating institutes have the biosecurity permissions to develop *in vivo* experiments with radiolabeled molecules. Alternatively, we have tried to detect the administered phytosterols in whole brain membrane fractions (obtained at 100,000g) by HPLC with UV detection, but we did not have enough sensitivity using two different methodologies.

Interestingly, Vanmierlo et al. (2012; PMID: 22279184) measured plant sterol concentrations in homogenates and detergent resistant membrane (DRM) fractions of 3-month old C57BL/6 mice fed with phytosterols. These authors found that administration of phytosterols led to significantly elevated plant sterol concentrations mainly in DRMs regions of the brain membranes. Although phytosterols’ distribution was not measured in cochlear hair cells in that work, these data strongly support the idea that phytosterols are able to cross the blood brain barrier and reach the proper cellular compartment where cholesterol is normally enriched.

***-       Lines 372-373: "…with an apparent recovery of the normal prestin distribution in the lateral wall." Can you show the quantification of prestin intensity in the periphery of the cell?***

Prestin levels in OHCs from control, efavirenz-treated and efavirenz plus phytosterols-treated mice were evaluated by immunohistochemistry. We observed a reduction in prestin labeling in the lateral wall of ears treated with efavirenz compared to controls. Phytosterols supplementation in the diet increased prestin levels in the OHCs membrane compared with efavirenz treated mice leading to a partial recovery of the normal prestin distribution, as seen in the representative confocal images of whole mounts of the organ of Corti from the medial region on Figure 5A. To quantify the prestin intensity in the periphery of the cell as well as in the cytoplasm, we measured the pixel intensity along the length of three OHCs from each row from all the sections that were imaged at the confocal microscope from the three groups of mice. We measured the prestin intensity as a function of the distance by measuring in groups of three consecutive cells.

However, as the reviewer suggested, to have a better appreciation of the difference observed in the lateral wall compared to the cytoplasm, we have now changed this analysis by tracing the line on individual OHCs. The new Figure 5B shows a representative plot of prestin intensity at the medial region of the cochlea along the length of one OHC from all the sections that were imaged at the confocal microscope from the three groups of treated mice. The pink and orange colored line represents 8 μm, the average diameter of a single OHC. As shown in Figure 5B, pixel intensity values between the region corresponding to 0-2 μm and 6-8 μm represent the approximate width of the lateral wall of one individual OHC (indicated by the pink color of the line) and the orange part of the line indicates the cytoplasm of the OHC. Prestin intensity plot in the areas corresponding to the lateral wall showed a reduction in pixel intensity in the efavirenz treated group compared to controls and a slight, but significant, increase in the prestin signal after phytosterols supplementation. We have now quantified the change in the distribution of prestin within each OHC by calculating the ratio in the mean fluorescence intensity in the membrane area and the cytoplasm and showed the results in Fig 5C. In the control condition the ratio rendered a value of 1.20 ± 0.09 (n = 24 OHCs from 6 mice), indicating a higher presence of prestin in the cell membrane compared to the cytoplasm. In efavirenz treated animals this ratio dropped to 0.74 ± 0.04 (n = 25 OHCs from 6 mice) showing a higher presence of prestin in the cytoplasm compared to the membrane. This effect was partially reverted in the animals treated with phytosterols in which the ratio was 1.09 ± 0.03 (n = 38 OHCs from 6 mice). We have now added this information in the Results section of the new version of the manuscript and in the new Figure 5.

***-       Why were only male mice used in the study?***

We recognize the importance of testing female mice. Taking into account that cholesterol is the main precursor for several sexual hormones, we considered that during the aging period (2 to 24 months) female mice would initiate the typical fertility decay due to hormonal changes at 8 months of age, introducing an extra variable whose influence would be hard to interpret in the context of the cochlear changes induced by age.

In any case, we need to expand our studies to BALB/cJ male mice treated with efavirenz and/or phytosterols, and we will definitely conduct hearing experiments in female C57BL/6 and/or BALB/cJ mice in the near future.

***Some other minor points:***

***-       Could you enhance the contrast/brightness of calretinin staining in Fig 1?***

As the reviewer suggested we have now enhanced the contrast/brightness of calretinin in Figure 1.

***-       Could you also include ABR and DPOAE waveforms?***

As the reviewer suggested, we have now included the ABR and DPOAEs waveforms as a new Figure 3 in the revised manuscript.

-       ***Given that efavirenz produces dyslipidemia but also dysglycemia (doi: 10.1097/MD.0000000000002385), how do you know its effect is not due to the alteration on glucose metabolism?***

In the article by Sinxadi et al, 2016; PMID: 26765416, 99% of the patients included in the study were under treatment with stavudine in addition to efavirenz. It is true that higher plasma efavirenz concentrations were associated with higher plasma lipid and glucose concentrations, however the contribution of other antiretroviral drugs to this effect could not be excluded. On the other hand, in our study we used efavirenz concentrations 300-fold lower than the doses used for HIV treatment. Although we did not measure plasma lipid and glucose concentrations in efavirenz treated mice, we believe that changes in lipid levels in serum would not influence the results found in hair cells since peripheral cholesterol and triglycerides do not cross the blood-brain barrier (BBB). In the ear, it is called the blood-labyrinth barrier (BLB) and it is analogous to the BBB, which plays a critical role in limiting the entry of inflammatory and infectious agents into the central nervous system. For the case of glucose levels, only extremely high glucose concentrations maintained by prolonged periods was shown to affect hair cells’ function. Even if -at the low concentrations of efavirenz used- glucose levels in serum increase, we do not expect an effect of glucose in the electrophysiological parameters measured in our work.

***-       Does the treatment with efavirenz change the level of CYP46A1 levels in the cochlea?***

It has been shown that efavirenz enhances the activity of CYP46A1 both *in vitro* and *in vivo* (Mast, N et al. 2014; PMID: 24352658) but the levels of CYP46A1 after efavirenz treatment were not measured in the work. Studies performed to analyze the transcriptional regulation of the *cyp46A1* gene have shown that the expression of this gene is only responsive to increased oxidative stress and proinflammatory cytokines (Ohyama, Y et al. 2006; PMID: 16321981). *cyp46A1* gene expression was shown not to be responsive to changes in cholesterol or hydroxysterol levels. Finally, there is no data showing a post-transcriptional regulation of CYP46A1. For these reasons, we believe that CYP46A1 levels would not change with efavirenz exposure.

**Reviewer #3:**

***Numerous studies have shown cholesterol imbalances in the development of neurodegenerative diseases, but the role of cholesterol in the physiopathology of the inner ear has not been studied. This paper characterized cholesterol homeostasis in the inner ear of young and old mice, and tested the hypothesis that cholesterol deficiency might play a role in cochlear pathologies and hearing loss by analyzing CYP46A1 levels and cholesterol content in the inner ear of young and aged mice. The authors also tested the effects of efavirenz on the auditory function. The effect of dietary supplementation with phytosterols was also tested in the inner ear of mice treated with efavirenz. Overall, their findings are new and interesting, but no histological examinations of cochlear cells/microstructures (e.g., hair cell counts, spiral ganglion neuron/auditory nerve fiber density, stria vascularis thickness, ribbon synapse counts, etc.) was performed, although the aim of the study was to test the hypothesis that cholesterol deficiency might play a role in cochlear pathologies and hearing loss. Cholesterol levels were not measured in the whole inner ear tissues from the experimental groups (young, old, control, efavirenz, and efavirenz + phytosterol groups). In addition, I have some questions about the age of the old group and the statistical method. I elaborate on these and other points below.***

We thank the reviewer for acknowledging the fact that our findings are new and interesting. ***Line 94.  The authors stated that the aim of this study was to test the hypothesis that cholesterol deficiency might play a role in cochlear pathologies and hearing loss, but no histological examination of cochlear cells/microstructures (e.g., hair cell counts, spiral ganglion neuron/auditory nerve fiber density, stria vascularis thickness, ribbon synapse counts, etc.) was performed in the young, old, control, efavirenz, and efavirenz + phytosterol groups. So I don't think the proposed hypothesis was thoroughly tested in the current study, and the overall finding seems inconclusive in terms of testing the hypothesis.***

As shown in Figures 1 and 2, as well as in the new supplementary Figure 1, we performed two types of histological studies from acutely dissected whole mounts organ of Corti in young and old mice: 1) immunostaining against CYP46A1 and calretinin (to label sensory cells) and 2) staining against filipin and phalloidin (to label the cilia of sensory cells).

We have now included in the new Supplementary Figure 1, a bar graph showing the percentage of OHCs survival in aged mice compared to young animals. At two years of age, there was a 70% of OHCs degeneration along the whole length of the cochlea. It is important to note, that at two years of age, in this C57BL/6J strain of mice, all the sensory epithelia is damaged and mice are completely deaf. As seen in Figure 1 by the calretinin immunostaining, IHCs also show an abnormal shape, however, damage to the OHCs was more prominent than the damage to IHCs.

Multiple studies in C57BL/6J mice have linked the typical age-related loss in this strain with the degeneration of the whole sensory inner epithelium due to loss of OHCs and the primary auditory neurons, the spiral ganglion neurons. Thus, it has been well described in the literature that hair cell and ribbon synapse counts, spiral ganglion neuron/auditory nerve fiber density is reduced during aging in C57BL/6J mice (Willott *et al*., 1995; PMID: 8575990; Parham, 1997; PMID: 9367243; Fetoni *et al*., 2011; PMID: 21211561; Alvarado *et al*., 2014; PMID: 24634657; Möhrle *et al*., 2016;PMID: 27318145).

In the case of the groups of young animals treated with efavirenz and efavirenz + phytosterols, as shown in Figures 4 and 5, as well as in the new supplementary Figure 2, we performed two types of histological studies from acutely dissected whole mounts organ of Corti in controls and drug-treated mice: 1) staining against filipin and phalloidin, to evaluate cholesterol levels and identify hair cells, respectively, and 2) immunostaining against prestin, to evaluate the prestin expression levels in the lateral wall membrane of OHCs, and calretinin as a hair cell marker. With these two types of histology techniques we could easily detect that the sensory epithelium from controls and treated-groups was grossly normal: no hair cell or supporting cell loss.

In Figure 3 we show that there was an elevation in DPOAEs thresholds together with a slight elevation in ABR thresholds. Moreover, we observed that there was no alteration in suprathreshold ABR peak 1 amplitudes at different test frequencies. This fist peak of the ABR wave represents the summed sound-evoked spike activity at the first synapse between IHCs and auditory nerve fibers (the so-called ribbon synapses). Normal cochleae display linear rises in ABR peak 1 amplitudes in response to increasing sound stimulus whereas neuropathic cochleae do not (Kujawa & Liberman, 2009; PMID: 19906956). As shown in Figure 3C, there was no alteration in the mean ABR peak 1 suprathreshold amplitudes in all the groups, indicating that there was no cochlear synaptopathy, i.e. loss of ribbon synapses or changes in spiral ganglion neuron/auditory nerve fiber density. Taking into account that the physiological data pointed to deficits in cochlear amplification rather than synaptic transmission, and considering that we had a limited amount of tissue per group to perform histology, we decided to focus on detecting cholesterol changes and alterations in OHCs. We don’t dismiss that structural changes not evident in physiological measurements could be present and their analysis will be considered in further studies.

***Method.  I understand cholesterol levels were measured using the cholesterol-binding fluorescent antibiotic filipin in the fixed hair cells, but I do think total cholesterol levels should also be measured in the whole inner ear tissues of all the experimental groups (young, old, control, efavirenz, and efavirenz + phytosterol groups) to test the hypothesis that cholesterol deficiency might play a role in cochlear pathologies and hearing loss.***

In previous works we have shown that CYP46A1-triggered cholesterol loss is detectable in specific cell populations but it is missed when total cholesterol is measured in whole tissues. In mice, aging-triggered cholesterol loss is observed only in hippocampal neurons but not when extracts of the whole brain are analyzed (Martin et al., 2008; PMID: 18287532). This is because CYP46A1 expression seems not to be regulated in the same way in every cell population. Furthermore, different cell populations have different capabilities to synthesize cholesterol. For example in the brain, cholesterol is actively synthesized by astrocytes whereas low levels of cholesterol are synthesized by neurons (Pfrieger and Ungerer 2011;PMID: 21741992). In fact, CYP46A1 activity leads to decreased cholesterol levels in neurons but not in astrocytes. For that reason the rationale behind the methodology selected for this work was that cholesterol loss in the different cell populations of the cochlea would be better assessed by filipin staining of the different cell types, instead of measuring total cholesterol levels in the whole inner ear.

***Line 109.  C57BL/6J mice develop early onset severe and progressive hearing loss by 12-15 months of age, with the disruption of both outer and inner hair cells, due to the Cdh23Ahl allele (***[***https://www.jax.org/strain/000664***](https://www.jax.org/strain/000664)***), so it is not clear to me why 24 months old mice were used when they were near deaf and likely lost most of the hair cells. I think use of 3 age groups (e.g., 2, 6, 12 months old groups) or 2 age groups (e.g., 2, 12 months old groups instead of 2 and 24 months old) would make more sense for the measurements of CYP46A1 and filipin labeling.***

The C57BL/6J is the most widely used strain for aging studies in the central nervous system (CNS) since they are able to survive for up to 27 – 29 months of age. Most of the studies about alterations of cholesterol homeostasis in aged-mice were performed in C57BL/6J mice. Using this strain, we have shown that aging increases CYP46A1 levels in the hippocampus of 24 month-old mice (Martin et al., 2014; PMID: 24878762) leading to cholesterol loss and cognitive decline. These deficits, at least in the hippocampus, are not observed before the mice reach the age of 19 months. That was the reason to explore the CYP46A1 levels at an advanced age, like 24 months, in C57BL/6J mice. The reviewer is right that it would be a good idea to analyze CYP46A1 levels in hair cells at earlier times (i.e. at 12 months). However, when we measured the CYP46A1 mRNA at 12 months for this revised version of the manuscript, we found that the relative expression levels of CYP46A1 were similar in young and 12-months old mice, similar to that previously reported in the aging hippocampus (Martin et al., 2008; PMID: 18287532). As it was stated above, a large number of CNS changes induced by aging in C57BL/6 mice start to be evident once the animals reach the 22 months of age (as example see Lemaitre et al., 2023; PMID: 36975362). This is the reason why the usual experimental time point for aged C57BL/6 mice is 22-24 months.

As part of a future project, we are planning to use other strains like CBA/CaJ or BALB/cJ mice that do not have an early onset of age-related hearing loss.

Even so, we consider that our present work is the first proof-of-principle showing that efavirenz produces hearing deficits because of its action in cholesterol removal from OHCs leading to alterations in the function of the motor protein prestin. Moreover, we show that the effect of cholesterol loss in OHCs can be rescued with phytosterols supplementation in the diet.

We have now added this issue in the Discussion section in the new version of the manuscript.

***Fig. 3/Fig. 8.  I am not sure how this can be Kruskal-Wallis since (I think) the factors are 3 experimental groups and frequencies. My understanding is that Kruskal-Wallis cannot handle two way ANOVA data in the non-parametric sense. Could you clarify this?
In addition, is there any reason 6 of each group was used for the ABR and DPOAE tests?***

For the statistical analysis of the auditory physiology data, the available animals (18 young animals) were randomly distributed in three groups corresponding to the following treatments: Control n=6; Efavirenz n=6 and Efavirenz + Phytosterols n=6. In this way, three independent groups of animals were formed, each one corresponding to a level inside the treatment factor. In our experimental design, the frequency is not considered as another factor because the different pure tones at different test frequencies are presented in a continuous way along a specific time in all the groups. If frequency were treated as a factor there should be a group of independent animals for each frequency, which is not the case in our experimental design. All animals were tested with all frequencies. For this reason, groups that are independent were compared with a nonparametric test such as Kruskall Wallis.

***Line 158.  The aim of this experiment was to test the hypothesis that CYP46A1 activation might reduce the cholesterol content in young mice, leading to hearing deficits. So, I think histological examination of cochlear cells (such as hair cell counts) should also be performed at the end of the treatment (at 3 months of age) to validate the ABR test results. In addition, what is the rationale for starting efavirenz treatment at 2 months of age for just 4 weeks as opposed to starting at a later time point (e.g., 5 months of age) for a longer period (e.g., 3 months)?***

At the end of the treatment, we did not observe any hair cell loss by histological examination of whole mounts organ of Corti using antibodies against calretinin and prestin that label both IHCs and OHCs, respectively. We have now added this in the new version of the manuscript.

The rationale for starting efavirenz treatment at 2 months of age for 4 weeks is because the C57BL/6J mouse strain displays the classic pattern of age-related hearing loss starting at 8-10 months of age (Someya S. et al., 2009; PMID: 19901338). It is well established that C57BL/6J mice at 2 months of age have a good hearing sensitivity by ABR and DPOAEs recordings (Kikkawa Y. et al., 2012; PMID: 22531723; Parham K., 1997; PMID: 9367243). If we had started the treatment at a later time point (e.g., 5 months of age) for a longer period (e.g., 3 months) we would have had the effects of the early onset and progressive hearing loss characteristic of this strain.

***Lines 231 and 265.  Again, what is the rationale for starting efavirenz + phytosterol treatment at 2 months of age for just 3 weeks as opposed to starting at a later time point (e.g., 5 months of age) for a longer period (e.g., 3 months)?***

As stated above, the rationale for starting efavirenz + phytosterols treatment at 2 months of age for 4 weeks is because the C57BL/6J mouse strain displays the classic pattern of age-related hearing loss starting at 8-10 months of age (Someya S. et al., 2009; PMID: 19901338).

***Fig. 4, 5, 9, 10 (bar graphs).  I think error bars should be added to the bar graphs.***

The error bars are present in the graphs but they are very small because of the large number of analyzed cells.
