# Supplementary material for: Phytosterols reverse antiretroviral-induced hearing loss, with potential implications for cochlear aging
Source: PLoS Biol. 2023 Aug 24;21(8):e3002257. doi: 10.1371/journal.pbio.3002257 (PMC10449472; doi:10.1371/journal.pbio.3002257)
Supplement: Attachment — Submitted filename: Response to Reviewers.docx [file pbio.3002257.s004.docx]

**Revision**

**Title: "Phytosterols reversal of antiretroviral-induced hearing loss and its implications in presbycusis"**

**PBIOLOGY-D-22-01971R2**

**Authors: Alejandro O. Sodero, Valeria C. Castagna, Setiembre D. Elorza, Sara M. Gonzalez-Rodulfo, María Alejandra Paulazo, Jimena A. Ballestero, Mauricio G. Martin and María Eugenia Gomez-Casati**

**Editor's Remarks to Author:**

***The reviewers largely agree that the manuscript has been strengthened in the revision, however Reviewers 1 and 2 have a number of lingering concerns and additional comments that we think should be addressed. In some cases, Reviewer 2 has requested additional data. While adding this data would strengthen the study, after discussion with the Academic Editor, we think this would not strictly be required for publication at PLOS Biology. However, in the absence of new data, you should address Reviewer 2's concerns by adding a section to the discussion outlining some of the methodological considerations and limitations of the study.***

We would like to thank the PLOS Biology Editors and the Academic Editor for giving us the opportunity of further revising our work and the reviewers for their helpful comments.

We have reworked the manuscript to fully address the reviewers’ suggestions. The manuscript has been changed accordingly and a point-by-point response to the comments raised by Reviewers 1 and 2 is included. We have now introduced a paragraph discussing the methodological considerations and limitations of the study.

***In addition to addressing the reviewer comments, below, we also ask that you please address the following editorial requests:***

***1) TITLE: We think that the title should be edited slightly to avoid punctuation and for clarity. If you agree, we suggest you change it to something like "Phytosterols reverse age-related cochlear changes and hearing impairments caused by antiretroviral treatment in mice"***

Thanks for your suggestion. Taking into account that in the present work we did not directly demonstrate that phytosterols can reverse age-related cochlear changes, to avoid punctuation and for clarity we have now edited the title and we propose “*Phytosterols reversal of antiretroviral-induced hearing loss and its implications in presbycusis*”.

***2) ARTICLE TYPE: After discussion within the team, we think that your manuscript is closest in scope to our 'Short Report' article type, and so we request that you change it accordingly. Short Reports can have a maximum of 4 figures and so this will require that you condense 2 of your figures or move one to the supplement. We suggest that Figures 1 and 2 might be combined to reduce the figure number to 4. For more information about PLOS Biology Short Reports, see here: https://journals.plos.org/plosbiology/s/what-we-publish#loc-short-reports***

We have now changed the manuscript and figures accordingly to a “Short Report” article type. As editors suggested, we have reduced the number of figures to 4 by combining Figures 1 and 2. All the supplementary information remains unchanged.

***3) DATA: Thank you for providing the underlying data for your study as a deposition on OSF. Can you please add a sentence to each figure legend (including supplemental) referencing this dataset? For example, you can add the sentence "the data underlying this figure can be found at https://osf.io/xpemk/"***

We have now added a sentence to each figure legend (including supplemental) referencing the underlying dataset on OSF.

**Reviewer Comments:**

**Reviewer #1:**

***In the revised version of the manuscript "Phytosterols reverse hearing impairments caused by antiretroviral treatment. Implications in presbycusis" the authors have addressed most of my previous comments.***

***However, I still have a few comments that should be addressed.***

We thank the reviewer for acknowledging the fact that we have addressed the questions raised in the previous version. As the reviewer recommended, we have performed changes accordingly throughout the manuscript.

***1)	You report group differences by stating the significance found in the ANOVA, please also include the results from the post-hoc test.***

Thanks for your suggestion; we have now added the results from the post-hoc test in all the cases that we have applied the ANOVA test throughout the manuscript.

***2)	I am not sure what happened, but all the figures are blurry. The supplemental material is not blurry. I would recommend that you use that format throughout the manuscript.***

We are very sorry for that. Something must have happened when figures were converted to the pdf file. We have now verified all the figures and checked they are all in a high resolution format. We are now uploading high quality images in both TIFF and EPS file formats.

***3)	In Fig 3 you give a scale bar of 50 dB. Please give the reference if that is dB SPL or something else.***

The scale bar in the new Figure 2 (previous Fig 3) is 50 dB SPL. We have now added it in the new Figure.

***4)	When you plot distance and the pink dotted line (e.g. Fig 4B) it is not clear immediately that the pink dashed line is representing the dashed line from panel A. Maybe instead of assigning it a level on the y-axis put it underneath the x-axis? That might also explain the 'distance' measurement a little better. Or maybe use the term width? In the figure caption the term length is used. Please make sure you keep the terms consistent throughout the manuscript.***

"As suggested by the reviewer, we have made changes to the new Figure 3. Specifically, we have placed the pink dotted line underneath the x-axis to more accurately represent the line shown in panel A. This line corresponds to the location where the quantification of filipin was performed in two adjacent OHCs." We have now changed “length” for “width” to keep the terms consistent throughout the manuscript. We have done the same changes in the new Figure 4 and now the pink and orange line is underneath the x-axis.

***5)	Also in Fig 4, please explain the pink dashed lines already for A and not only in B.***

We have now explained the pink dashed line in the new Figure 3 for panel A, as well.

***6)	Page 26 line 645 and 646: I think you mean 0-2 and 6-8 and 2-6 not 2-4.***

Thanks for noticing this mistake. We have corrected that in the new version of the manuscript.

**Reviewer #2:**

***In the revised version of the manuscript, Sodero et al. did not provide alternative methods for CYP46A1 quantification, so I am still not convinced that CYP46A1 increased expression is one of the mechanisms behind ARHL. Also, quantification of cholesterol using filipin staining in an intact (young) and a degenerated (aged) tissue makes comparisons between groups unreliable (I will elaborate on that). As the authors mentioned, they are exploring the effect of aging and efavirenz administration in cholesterol levels in the cochlea using other strains of mice, which would make the results stronger. Determination of a dose-response curve for the effect of efavirenz in the cochlea will be fundamental to understand the effect this drug has on patients. I do believe that the efavirenz results are very interesting since they show that localization of prestin in OHCs is affected by the drug administration and reverted by phytosterols consumption, which could explain the DPOAEs thresholds elevations and recovery respectively.***

We thank the reviewer for the comment about the fact that efavirenz results are very interesting. Although we do recognize the limitations of the techniques used in this work (we will address them specifically below) we feel that we present converging information that supports our conclusions. Here we found that an increase in the cholesterol hydroxylating enzyme CYP46A1 in cochlear aged tissue, measured by immunofluorescence, correlates with a decrease in cholesterol content at the same age by filipin staining (as we comment below, we included another way to quantify filipin staining in young and old OHCs to avoid possible artifacts due to a difference in cell orientation). Afterwards, we used the anti-HIV medication efavirenz to enhance CYP46A1 activity and we observed a decrease in cholesterol content in young mice leading to hearing deficits. These results strongly support the cause-effect relationship between CYP46 increased activity and hearing loss.

We are aware that there are very important questions still open like the determination of the dose-response effects of efavirenz in the cochlea. This question remains fundamental to explore in our future project, and it is crucial to translate the results of this study into a viable treatment option for patients. However, the fact that efavirenz has dual effects on CYP46A1 activity (activator at low doses and inhibitor at higher doses (Mast N. et al., 2014; PMID: 24352658), indicates that having a proper assessment of dose dependency requires a thoughtful study that we consider is out of the scope of the present work, intended as a proof of concept.

Another important question left is whether phytosterols' supplementation can delay and/or ameliorate the cochlear changes triggered by aging. We are currently studying this phenomenon in other mouse strains in order to confirm that the observed effect is not related to the early onset of hearing loss that the C57BL/6 line presents. For this future project, we are willing to include an alternative method for CYP46A1 quantification as RNAscope or single cell RNAseq to reinforce our immunofluorescence studies.

***Some points to work on are:***

****	Figure 1: According to the immunostaning data, the CYP46A1 intensity level is very different in the organ of Corti of aged vs young mice and this is not just in hair cells so the fact that it was not detected through qPCR makes the staining data less reliable, even though the ages were not exactly matched with the ones shown in the paper. The authors should perform RNA Scope (or other quantitative in situ hybridization method) to resolve the discrepancy in an age where they detect the decrease in protein level in a cell-specific manner.***

We understand the fact that the absence of differences in a whole cochlea qRT-PCR might shed doubts into the immunofluorescence results. However, it is important to note that the cochlea consist of a complex array of different cell types with at least four distinct functional domains: sensory epithelium with hair cells and supporting cells; neuronal compartment; lateral wall and immune cells (Milon B et al., 2021; PMID: 34592158). Because of the cellular diversity of each of the functional subdomains of the cochlea, sensory cell changes in gene expression are less likely to be detected using RNA extracted from an intact auditory epithelium. Even though we show an increase in immunostaining that looks generalized in old mice, it is worth noting that we are only showing a small portion of the cochlear tissue (the sensory domain). Moreover, our final hypothesis is that the effect of efavirenz is related with changes that occur in a specific hair cell type, the outer hair cells. Thus, the relevant information for the hypothesis is the changes in the expression at a single cell level.

We agree with the reviewer that the work would benefit from utilizing a cell-type-specific approach, such as single-cell RNA-seq (scRNA-seq), or quantitative staining procedures such as RNA scope and we are planning to engage these types of analyses for future studies. However, for this first work we feel that immunostaining, although semiquantitative, does provide reliable information regarding changes in expression level. In fact, immunostaining is the standard validation method for protein expression after *in situ* RNA expression assay by RNA-scope in cochlear tissue providing consistent results (	Herzano R, et al. 2020; PMID: 32579737 and Sun S, 2018; PMID: 30078710).

We have now added a section in the new version of the manuscript discussing the limitations of present study and future directions.

****	Figure 2: In the case of young animals, OHC are oriented more perpendicular to the plane of the photo, but this is not the case for aged animals since there is substantial hair cell death and the orientation of the cells becomes less defined. This makes comparisons between groups very inconsistent because the cell volume that is being included in the quantification zone is larger for the young group than for the aged group.***

We agree with the reviewer that in the confocal pictures of filipin stained cochlear whole mounts, OHCs are oriented more perpendicular to the plane of the photo in young ears. In aged animals, where there is substantial hair cell degeneration, the orientation of the OHCs becomes less defined. At two years of age, the cochlear tissue of the C57BL/6J strain has a high percentage of OHCs loss along the whole length of the cochlea as well as changes in IHCs and SCs morphologies together with some IHCs loss. Thus, it is normal to see a substantial disorganization of cochlear tissue during aging and the reviewer is right that this could lead to inconsistency when fluorescence quantifications are compared between groups. To analyze and compare cochlear tissue in young and aged mice, we performed the quantification of filipin fluorescence intensities by two different methods to be sure that the changes we observed are consistent between these two groups:

1) In the first method, we draw a line through the middle of every individual cell in each z-stack projection and fluorescence intensity was quantified along this line in young and aged tissue. This quantification method was already described by (Rajagopalan L. et al., 2007; PMID: 17933870) and was the method that we chose to use in the present manuscript in order to be consistent with the literature.

2) In the second method, we select an area (ROI) delimited by the cell boundaries and compare the integrated density of the fluorescence signal in whole individual cells in each z-stack projection in young and aged tissue. We found the same difference between young and aged tissues, as with the method described in the present manuscript.

The values obtained with this method were: 296.9 ± 13.2 in young (n = 3 animals; 200 OHCs) and 34.4 ± 0.8 in aged (n = 6 animals; 22 OHCs) and 415.7 ± 15.1 in young (n = 3 animals; 104 IHCs) and 64.8 ± 4.9 in aged (n = 6 animals; 52 IHCs) (Arbitrary units mean ± SEM, t-test, p <0.0001 in both cell types).

Thus, we consider that the difference in filipin fluorescence intensity observed in OHCs of whole mount cochlear tissue obtained from young and aged mice (more than 85% of reduction in filipin staining by the two different methods of quantification) is fairly consistent.

****	Figure 3: How did the authors call the ABR thresholds? If manually done, was it done by more than one person? Was this done blindly? An algorithm can be used for that (DOI: 10.1016/j.heares.2019.107782). In the efavirenz + phytosterols traces, it looks like another person or an algorithm could very likely pick the intensity immediately bellow the one called by the authors since there appears to be small but clear peaks 1 & 2.***

The ABR threshold was determined by visual inspection by two different experimenters that were blind to the animal treatment condition to avoid introducing experimenter biases in the group comparisons. Threshold for ABR was defined as the mean between the lowest stimulus level at which a repeatable peak 1 could be identified in the response waveform and the sound intensity before that.

In the Materials and Methods section in the new version of the manuscript we have included that the ABR threshold determination was done offline blindly by two different experimenters.

In the efavirenz + phytosterols blue traces in the new Figure 2A, the reviewer says that another person or an algorithm could very likely have picked the intensity immediately below 30 dB SPL, because there appears to be small but clear peaks 1 and 2. In this particular case, we consider that at 30 dB SPL the signal-to-noise ratio was very small to identify a measurable peak. While we are recording, the PXI system automatically graphs the peak-to-peak amplitude of the averages ABR (in microvolts) obtained at each tone pip SPL together with the noise floor, i.e. the peak-to-peak amplitude at subthreshold levels. In this way we can determine the ABR thresholds by comparison with the stacked waveforms at all tone pip levels.

Although over the past decades numerous attempts have been taken to perform automated and objective assessment of ABR thresholds, no general gold standard has been found applicable in all laboratory settings. Moreover, implementation of such algorithms typically requires a significant amount of troubleshooting and optimization and may only be reliable in a narrow range of laboratory conditions (Suthakar K, et al., 2019; PMID: 31437652). The main limitations are differences in signal-to-noise-ratio, conditions, electrode placement/impedance, as well as acquisition settings. This makes the accurate threshold determination only possible under a narrow range of experimental settings, which hampers direct comparisons of ABR results across different laboratories (Wang H, et al., 2021; PMID: 34765914). In the absence of a universally accepted technique, visual inspection is an accepted method for threshold determination in the field.

***I agree with lines 540-545 where the authors mention that it is relevant to study this phenomenon in other mouse strains in order to confirm that the effect seen is not related to the early HL that the C57BL/6 line presents. In that sense, I believe that including an alternative method for CYP46A1 quantification will be important to make the immunostaining results more reliable, together with an antibody that can label OHC homogeneously across ages (like Myo7a).***

We agree that it is very relevant to perform this study in other mouse strains. In fact, we already started a project on aging BALB/cJ mice (until 1-2 years) to analyze if phytosterols supplementation can delay the onset of presbycusis. Moreover, we are planning to analyze more deeply the dose dependency of efavirenz in different mouse strains (C57BL/6J and BALB/cJ) as part of coming projects.

As the reviewer suggested, we will definitely include an alternative method for CYP46A1 quantification to make the immunostaining results more reliable, together with the Myo7a staining that labels OHCs homogeneously across ages. Nevertheless, we believe that our present work is highly relevant because it is the first proof-of-principle showing that CYP46A1 activation can lead to hearing deficits through cholesterol removal from OHCs, leading to alterations in the distribution of the motor protein prestin in the lateral wall membrane, all these having functional consequences. Moreover, we provide a possible intervention showing that the effect of cholesterol loss on prestin localization on OHCs can be rescued with phytosterols supplementation in the diet. We have now added a section in the new version of the manuscript discussing the limitations of present study and future directions.

***Reviewer #3: The authors have addressed all of my major concerns.***

We thank the reviewer for acknowledging the fact that we have addressed the major concerns from the previous version.
